# Relationships between lumbar inter-vertebral motion and lordosis in healthy adult males: a cross sectional cohort study

**DOI:** 10.1186/s12891-016-0975-1

**Published:** 2016-03-10

**Authors:** Alister du Rose, Alan Breen

**Affiliations:** Institute for Musculoskeletal Research and Clinical Implementation, Anglo-European College of Chiropractic, Parkwood Road, Bournemouth, BH5 2DF UK; Faculty of Science and Technology, Bournemouth University, Fern Barrow, Poole, BH12 5BB UK

**Keywords:** Spine kinematics, Fluoroscopy, Lordosis, Reliability, Agreement

## Abstract

**Background:**

Intervertebral motion impairment is widely thought to be related to chronic back disability, however, the movements of inter-vertebral pairs are not independent of each other and motion may also be related to morphology. Furthermore, maximum intervertebral range of motion (IV-RoMmax) is difficult to measure accurately in living subjects. The purpose of this study was to explore possible relationships between (IV-RoMmax) and lordosis, initial attainment rate and IV-RoMmax at other levels during weight-bearing flexion using quantitative fluoroscopy (QF).

**Methods:**

Continuous QF motion sequences were recorded during controlled active sagittal flexion of 60° in 18 males (mean age 27.6 SD 4.4) with no history of low back pain in the previous year. IV-RoMmax, lordotic angle, and initial attainment rate at all inter-vertebral levels from L2-S1 were extracted. Relationships between IV-RoMmax and the other variables were explored using correlation coefficients, and simple linear regression was used to determine the effects of any significant relationships. Within and between observer repeatability of IV-RoMmax and initial attainment rate measurements were assessed in a sub-set of ten participants, using the intra-class correlation coefficient (ICC) and standard error of measurement (SEM).

**Results:**

QF measurements were highly repeatable, the lowest ICC for IV-RoMmax, being 0.94 (0.80–0.99) and highest SEM (0.76°). For initial attainment rate the lowest ICC was 0.84 (0.49–0.96) and the highest SEM (0.036). The results also demonstrated significant positive and negative correlations between IV-RoMmax and IV-RoMmax at other lumbar levels (*r* = −0.64–0.65), lordosis (*r* = −0.52–0.54), and initial attainment rate (*r* = −0.64–0.73). Simple linear regression analysis of all significant relationships showed that these predict between 28 and 42 % of the variance in IV-RoMmax.

**Conclusions:**

This study found weak to moderate effects of individual kinematic variables and lumbar lordosis on IV-RoMmax at other intervertebral levels. These effects, when combined, may be important when such levels are being considered by healthcare professionals as potential sources of pain generation. Multivariate investigations in larger samples are warranted.

**Electronic supplementary material:**

The online version of this article (doi:10.1186/s12891-016-0975-1) contains supplementary material, which is available to authorized users.

## Background

Movement of the lumbar spine requires the participation of multiple segments and the relevant contributions of segments are a function of their own mechanical properties [1]. Aberrant spinal movement patterns are widely thought to be related to musculoskeletal pain and dysfunction [2–4], and as such they are used to inform surgical and conservative clinical decision making [1, 5–7], and as indicators of spinal stability [3, 8–10]. As a consequence of their wide variation in both low back pain and healthy populations however, the clinical importance of factors such as inter-vertebral range of motion (IV-RoM) remains unclear, and the identification of biomechanical factors that may contribute to low back pain, remains a challenge [11]. Information about how IV-RoM may interact with other biomechanical factors may therefore help provide a better understanding of how variations in lumbar inter-vertebral kinematics may affect prognosis and treatment outcomes.

The starting point for this should be the collection of detailed normative quantitative data with respect to in vivo inter-vertebral motion and morphologic parameters [12]. Quantitative fluoroscopy (QF) has been shown to be an accurate and reliable 2D method of doing this [11, 13, 14]. Recent technological advances have enabled the acquisition of 3D lumbar kinematic data in vivo [15], however it has been demonstrated that there is only minimal axial rotation and lateral bending associated with movements in the sagittal plane [16–19], and in terms of QF inter-vertebral measurements, out of plane motion of up to 10° does not significantly affect accuracy [20]. Therefore, the greater expense and dose associated with current 3D techniques against the clinical and research benefits, perhaps justify the use of 2D QF technology, particularly in the sagittal plane. Indeed, the investigation of spinal mechanical behaviour has been outlined as a priority for future QF research [21], which begins with the relationships between IV-RoM and other kinematic variables in healthy, pain–free control populations. Such normative information should provide insights into the possible biomechanical consequences of changes within each.

Previous dynamic studies using fluoroscopy have highlighted contrasting ranges and patterns of angular rotation between the upper and lower lumbar motion segments [12, 22–24], which make different contributions to movements such as sagittal flexion. There is also evidence to suggest that lordosis may relate to an individual’s spinal flexibility [25]. Indeed, a recent MRI study that investigated the intrinsic shape of the lumbar spine concluded that lumbar spinal shapes may be related to an individual’s risk of injury [26].

IV-RoM is the most common measure of inter-vertebral motion [11, 13, 27] and attainment rate (defined as the velocity with which IV-RoM is reached), has been identified as a reflection of intervertebral restraint [11, 28, 29]. Initial attainment rate is a refinement of this which measures the slackness of an inter-vertebral motion segment in its initial phase of rotation [30, 31]. This parameter has been shown to correlate with the dynamic neutral zone [32], and is therefore also believed to be of importance when considering the stability of motion segments.

Relationships between these and other kinematic and morphologic variables have not been previously explored. This study examined the relationships between IV-RoMmax at lumbar inter-vertebral levels from L2 to S1 and lordosis, initial attainment rate and IV-RoMmax at other lumbar spine levels during forward bending in healthy controls. It also assessed the intra and inter-observer repeatability of the QF measurement of IV-RoMmax and initial attainment rate.

## Methods

### Study design

This was a cross-sectional, laboratory based cohort study of the relationships between L2-S1 IV-RoMmax and lordosis, initial attainment rate and IV-RoMmax at other levels.

### Participants

The eligibility criteria for the study are shown in Table 1. Twenty male participants were recruited from the Anglo-European College of Chiropractic (AECC) student population over a 5 month period between May and September 2014. National Research ethics Service (NRES) approval was acquired (Bristol 10/H0106/65) and prior to the collection of data, written informed consent was obtained from each participant. A participant number of 20 was selected, as a sample size ≥ 12 has been recommended as sufficient for the precision around the measurement to be used in an exploratory study [33].

**Table 1:** Eligibility criteria

Inclusion	Exclusion
Males aged 20–40 years	Inadequate understanding of English
An ability to understand written information	Currently receiving treatment for osteoporosis
Willing to participate and able to give informed consent	A history of recent abdominal or pelvic surgery
Consent to General Practitioner being informed	A history of previous lumbar spine surgery
A BMI of <30	A BMI of >30
No history of low back pain that prevented normal activity for at least 1 day in the previous year	Any medical radiation exposure in the past year or exposure in the past 2 years with a dose greater than 8 mSv
	Involvement in any other ongoing research study

### Data collection and processing

**Fig. 1:**
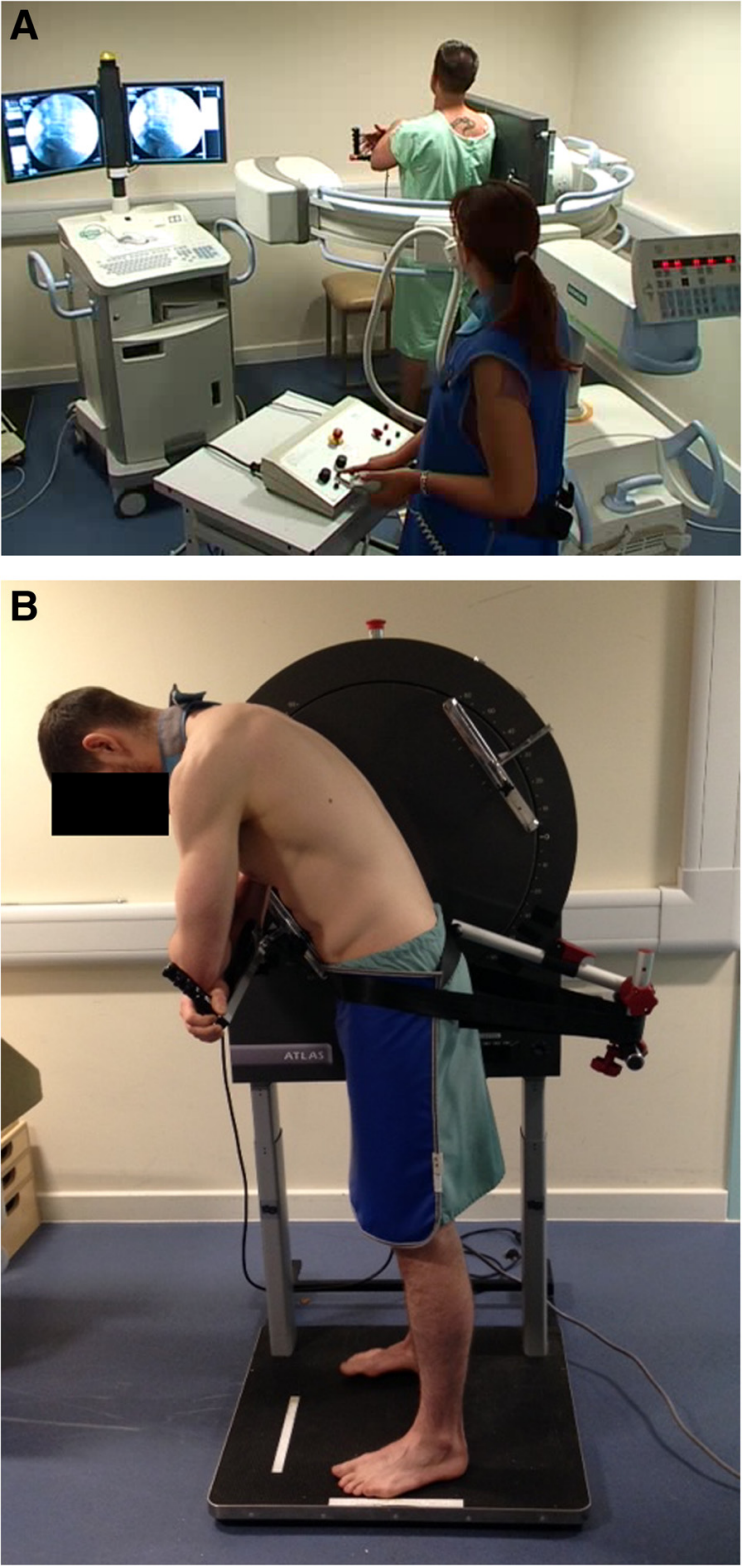
**a** Fluoroscope and weight-bearing motion frame. **b** Weight-bearing motion frame during flexion including pelvic restraint and lead protection

All data collection was conducted at the radiology department of the AECC. Fluoroscopic images of the lumbar spine were collected at 15 Hz using a Siemens Arcadis Avantic VC10A digital fluoroscope (CE0123) and a motion frame which acted to both stabilise the participants and guide their bending motion. Participants were asked to stand in a neutral upright position with their right side against the motion frame (Fig. 1a), and shadow a rotating arm rest which guided them during continuous fluoroscopic imaging, through a standardised range of 60° of forward flexion and return to upright, over a period of approximately 20 s. A review of spinal ranges of motion in controls proposed that the lumbar spine has an overall range (inclusive of both flexion and extension components) of approximately 80°, with 60° of this attributable to the flexion component [34]. It was therefore theorised that the majority of each participant’s lumbar inter-vertebral movement would be captured within this range.

Prior to image acquisition, participants were taken in 20° stages through to the full 60° to safeguard that they were able to tolerate the movement. The movement of the motion frame was recorded by electronic feedback from its motor drive, and synchronised with the fluoroscopic imaging. To minimise bending from the hip joints, the pelvis was stabilised using a strap secured around the anterior superior iliac spine bilaterally, and attached to an appendage of the motion frame directly posterior to the participant (Fig. 1b).

**Fig. 2:**
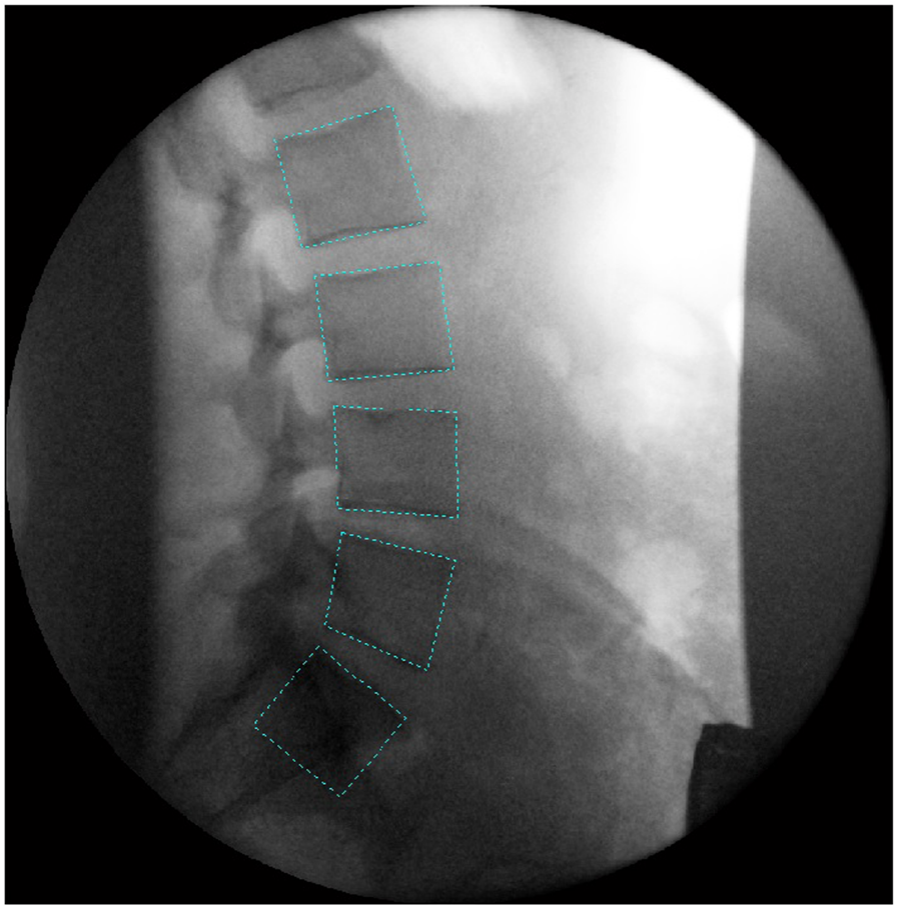
Fluoroscopic image of the lumbar spine. Templates placed around the lumbar vertebrae (L2-S1) on the first frame of the QF sequence

A lead apron was worn to shield the gonads, and participants were verbally reminded to maintain a neutral bending position during the flexion cycle. The position of the central ray was targeted at L4 to make sure that all vertebrae (L2-S1) were included in the image field Fig. 2.

The fluoroscopic sequences were then transferred to a desk top computer for analysis using bespoke image processing codes written in Matlab (The Mathworks, Cambridge). Using the screen cursor, the outlines of each vertebra from L2-S1 in the first image of each sequence were marked-up manually with an electronic template. In order to increase precision, this process was replicated five times for each sequence and the results were averaged. In all subsequent image frames the bespoke software tracked each vertebra automatically, creating a continuous measurement of its movement throughout the flexion and return bending sequence. To ensure that template tracking was maintained throughout the sequence, visual checks were made using video playback.

**Fig. 3:**
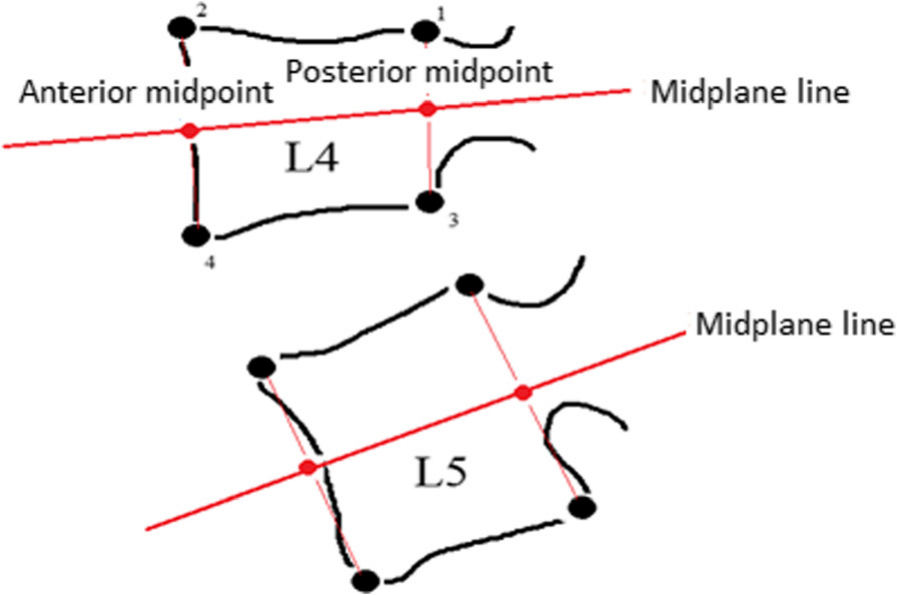
Frobin method to measure the change in inter-vertebral angle. Rotation is calculated as the angle between the two midplane lines

The data collected comprised of range of motion (IV-RoM), initial attainment rate, and lordosis and the reliability and agreement of the first two of these were assessed as part of the study [35]. The technique used to measure changes in inter-vertebral angle is discussed in detail elsewhere [36], and is shown in Fig. 3.

**Fig. 4:**
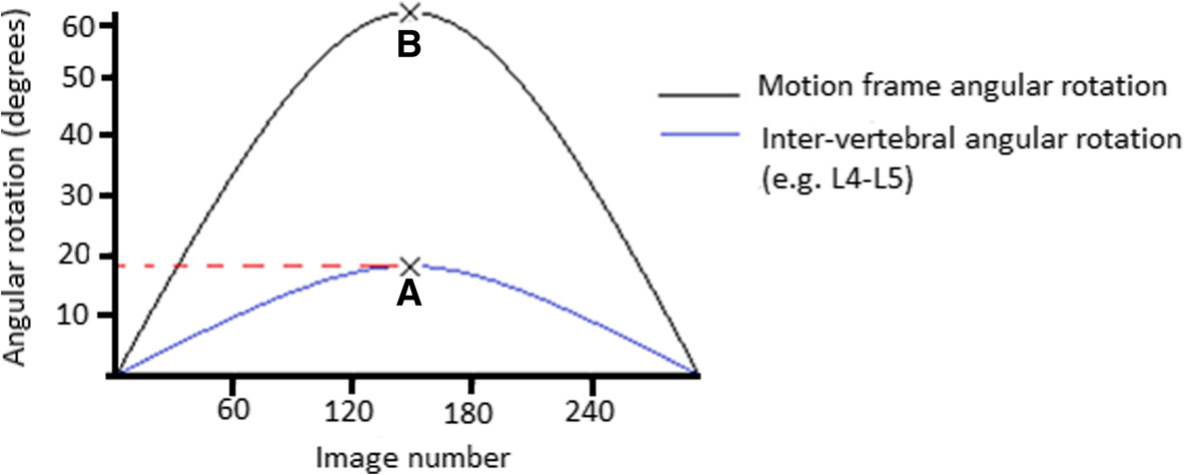
Calculation of the maximum angular range reached during flexion (IV-RoMmax). Maximum angle of rotation reached by each inter-vertebral motion pair (A); Maximum motion frame rotation (B) (always 60° during the QF sagittal flexion examination). Note: Maximum inter-vertebral range of motion may not always be found at the end of motion frame movement range

**Fig. 5:**
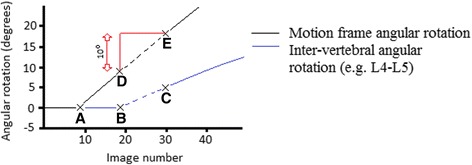
Calculation of initial attainment rate. The dotted lines represent the lines of best fit for motion frame movement (black) and inter-vertebral motion (blue), from which gradients can be calculated. Point at which the motion frame begins movement (A); Point at which inter-vertebral motion begins (B); Dotted line between (B) and (C) = the area under the curve from which the line of best fit is drawn to calculate inter-vertebral movement gradient; Dotted line between (D) and (E) = the area of the curve from which the line of best fit is drawn to calculate the motion frame movement gradient. Initial attainment rate is the calculated as the slope of BC/slope of DE

IV-RoMmax for each inter-vertebral level (L2-S1) was calculated as the maximum angular range reached at any point throughout the 60° flexion and return cycle (Fig. 4). Initial attainment rate for each level was calculated as the ratio of the slopes of motion frame movement and the inter-vertebral rotation over the first 10° immediately following the onset of inter-vertebral motion. The calculation of this variable has been outlined in detail elsewhere [31], and is also shown in Fig. 5. Lordosis was measured as the sum of all inter-vertebral angles (L2-S1), from the first image in the sequence. All participant data were anonymised.

### Reliability and agreement

A convenience sample of ten participants was used for the intra- and inter-observer repeatability studies. The intra-observer study was conducted, with a 6 week separation between image mark-ups. The inter-observer study images were processed by two independent observers. The first observer (template marker) was a medical physicist, and the second was ADR. The observers were blinded to the others’ results, and had 3 and 1 year(s) experience of template marking respectively.

### Data analysis

The normality of all data were tested using the Shapiro-Wilk test. Relationships between IV-RoMmax and other biomechanical variables, from normally distributed data were analysed using the Pearson product–moment correlation coefficient, and non-normal data using the Spearman’s Rank Correlation. Any significant relationships (*p* values < 0.05) were also analysed using simple linear regression. Intra- and inter-observer reliability and agreement of both IV-RoMmax and initial attainment rate measurements, were assessed using intra-class correlations (ICC 3, 1), and the standard error of measurement (SEM) respectively. Statistical analysis was performed using IBM SPSS (version 21).

Note: This study conformed to the STROBE checklist for reports of observational studies [37] (Additional file 1).

## Results

Twenty males satisfying the eligibility criteria consented to participate. Template tracking failure occurred in two participant’s sequences, and their data were removed. The mean (SD) age, height, and body mass Index (BMI) were 27.6 years (4.4), 1.8 m (0.06), and 24 (2.2) respectively. The average radiographic exposure factors for the group were documented as 79.7 kV SD (5.4) and 55.4 mA SD (3.4). ICRP103 conversion software PCXMC (Monte Carlo Simulation Package) was used to calculate the mean effective dose as 0.143 mSv.

### Reliability and agreement

#### IV-RoMmax

The ICC’s (reliability) and SEM’s (agreement) for both intra- and inter-observer IV-RoMmax studies are shown in Table 2. The results suggest excellent reliability with the smallest ICC being 0.96 (95 % CI 0.82–0.99) and 0.94 (95 % CI 0.80–0.99) for the intra- and inter-observer studies respectively. When comparing intra- and inter-observer repeatability, it was expected that intra-observer comparisons would demonstrate better reliability and agreement [11, 14]. This trend was not observed in these weight-bearing samples however, and ICC’s were the same or slightly better in the inter-observer group for two out of the four inter-vertebral levels. Agreement was found to be better than 1° at all levels, for both intra- and inter-observer studies.

**Table 2:** Intra- and inter-observer reliability and agreement of IV-RoMmax and initial attainment rate measurements during weight-bearing flexion and return *n* = 10

Inter-vertebral level	Intra-observer ICC (95 % CI)	Inter-observer ICC (95 % CI)	Intra-observer SEM (°)	Inter-observer SEM (°)
IV-RoMmax				
L2-L3	0.98 (0.92–1.0)	0.94 (0.80–0.99)	0.45	0.76
L3-L4	0.99 (0.96–1.0)	0.99 (0.67–1.0)	0.23	0.24
L4-L5	0.99 (0.97–1.0)	0.98 (0.93–1.0)	0.39	0.59
L5-S1	0.96 (0.82–0.99)	0.99 (0.94–1.0)	0.54	0.61
Inter-vertebral level	Intra-observer ICC (95 % CI)	Inter-observer ICC (95 % CI)	Intra-observer SEM_ratio_	Inter-observer SEM_ratio_
Initial attainment rate				
L2-L3	0.95 (0.78–0.99)	0.95 (0.80–0.99)	0.026	0.036
L3-L4	0.98 (0.92–1.0)	0.84 (0.49–0.96)	0.02	0.033
L4-L5	0.92 (0.71–0.98)	0.91 (0.70–0.98)	0.032	0.018
L5-S1	0.95 (0.81–0.99)	0.88 (0.53–0.97)	0.023	0.019

### Initial attainment rate

The ICC’s (reliability) and SEM’s (agreement) for both intra- and inter-observer weight-bearing initial attainment rate studies are also shown in Table 2. The reliability of initial attainment rate measurements was also acceptable, being more than 0.81 [38] in both intra- and inter-observer studies at all inter-vertebral levels. The smallest ICC was 0.84 (95 % CI 0.49–0.96) at the level of L3-L4 in the inter-observer study, and the largest was 0.98 (95 % CI 0.92–1.0) in the intra-observer study at the same level. The intra-observer study demonstrated consistently better reliability (including narrower confidence intervals) than that of the inter-observer study. The agreement of initial attainment rate measurements is also acceptable in both intra- and inter-observer studies. In the upper inter-vertebral levels (L2-3 and L3-4) SEM’s are comparatively lower in the intra-observer study, however in the lower levels (L4-L5 and L5-S1) SEM’s are comparatively higher.

### Correlations

A summary of the correlations between all biomechanical variables and IV-RoMmax is given in Table 3. Significant correlations were found between IV-RoMmax and at least one other variable at all inter-vertebral levels. These were consistently of mid-level strength (r - values ranging from −0.64 to 0.73). Lordosis was positively correlated with IV-RoMmax at L2-L3 and negatively with L4-5 (r = 0.54 and −0.52 respectively). In terms of IV-RoMmax versus IV-RoMmax at other levels, correlations were found between all levels except L5-S1. L2-L3 range was shown to be positively correlated with that of L3-4, but negatively correlated with L4-5. The strongest relationship was found between initial attainment rate at L3-4 and L5-S1 IV-RoMmax (r = 0.73). Indeed, initial attainment rate showed examples of strong correlations with range at all levels.

**Table 3:** Correlations between kinematic variables and IV-RoMmax at all inter-vertebral levels *n* = 18 (Significant relationships are highlighted in bold)

Kinematic variable	L2-L3 IV-RoMmax		L3-L4 IV-RoMmax		L4-L5 IV-RoMmax		L5-S1 IV-RoMmax	
	r	p	r	p	r	p	r	p
Lordosis	**0.54**	**0.021**	0.401	0.099	**−0.52**	**0.026**	−0.02	0.973
L2-L3 IV-RoMmax	-		**0.65**	**0.003**	**−0.64**	**0.004**	−0.35	0.157
L3-L4 IV-RoMmax	**0.65**	**0.003**	-		−0.29	0.234	−0.12	0.636
L4-L5 IV-RoMmax	**−0.64**	**0.004**	−0.29	0.234	-		0.15	0.558
L5-S1 IV-RoMmax	−0.35	0.157	−0.12	0.636	0.15	0.558	-	
L2-L3 Initial attainment rate	0.20	0.419	0.14	0.58	−0.09	0.713	0.21	0.403
L3-L4 Initial attainment rate	−0.18	0.465	−0.11	0.668	0.17	0.512	**0.73**	**0.001**
L4-L5 Initial attainment rate	**−0.53**	**0.023**	**−0.64**	**0.004**	**0.59**	**0.009**	0.02	0.949
L5-S1 Initial attainment rate	0.05	0.852	−0.02	0.938	0.07	0.776	0.42	0.079

### Simple linear regression analysis

**Fig. 6:**
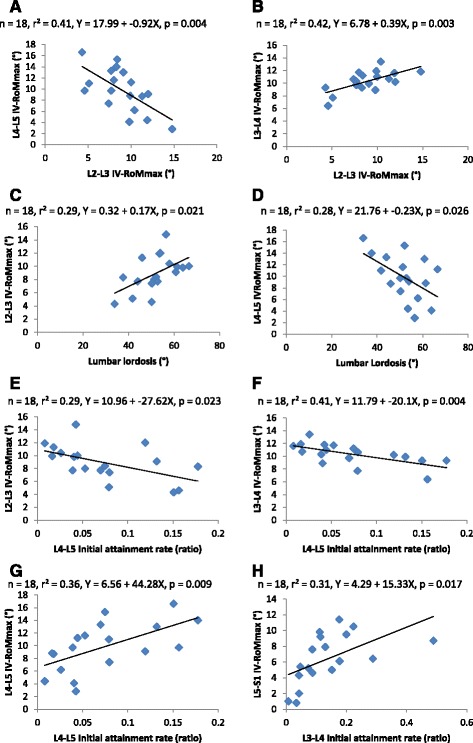
Scatter plots and linear regression values for all significant correlations. Significant relationships between IV-RoMmax and IV-RoMmax at other levels (**a** and **b**), lordosis (**c** and **d**), and initial attainment rate (**e**-**h**). *n* = sample size, *r* ^2^ = coefficient of determination, *Y* = linear regression equation, *p* = p value for the regression coefficient

The coefficients of determination (r^2^) for each of the significant correlations are shown in Fig. 6 (a-h). The values range from (0.28 to 0.42) and demonstrate that IV-RoMmax, can be influenced by lordosis, the IV-RoMmax at other lumbar levels, and initial attainment rate. Figure 6a for example shows that 41 % of the variability in L4-L5 IV-RoMmax can be accounted for by the range of L2-L3 IV-RoMmax.

## Discussion

### Agreement and reliability

The agreement and reliability of IV-RoM and initial attainment rate measurements using continuous QF image data has previously been assessed in recumbent participants [11]. As anticipated, the reliability and agreement of IV-RoM measurements during recumbent sagittal flexion were found to be similar to those found in the current work, with ‘substantial’ reliability [38], and acceptable error (i.e. <1°) demonstrated at all levels for both intra- and inter-observer studies. It has been demonstrated that reliability and agreement are typically decreased in the inter-observer group [11, 14], however these differences were shown to be minimal in the current study, and there were notable exceptions to the trend (Table 2). Although ICC’s were very similar between intra- and inter-observer groups, generally the width of the CI’s and the SEM’s were larger in the latter. It appears that whilst errors arising from the use of different observers did have a small impact, inter-observer agreement and reliability is still acceptable.

It was anticipated that there might be a marginal decrease in the repeatability of measurements at inter-vertebral levels closer to the edge of the image field (i.e. L2-3 and L5-S1). This predicted difficulty (due to superimposition of the ilia) in template marking/tracking of L5-S1 was the reason cited by Mellor et al. [11] for its exclusion, and it makes sense that tracking problems could be more likely to occur in templates that may partially leave the image field i.e. L2-3 and L5-S1. The current study’s results have shown however, that reliability and agreement of QF IV-RoMmax measurements at all levels, including L2-L3 and L5-S1, can be achieved in a weight-bearing protocol, at an acceptable level.

The initial attainment rate measurements were also highly repeatable; there was however a clearer distinction between the confidence intervals of intra- and inter-observer groups, being notably wider in the latter at L3-4 and L5-S1 levels (Table 2). These may be best explained by differences in the experience levels of the observers [14]. Differences in marking experience may also be the reason for the relatively increased measurement error in the inter-observer group at upper inter-vertebral levels, and improved agreement at the lower levels.

### Correlations

The results showed evidence of relationships between kinematic variables at multiple levels of the lumbar spine. IV-RoMmax at all inter-vertebral levels was significantly correlated, positively or negatively, with at least one other kinematic or morphological variable, and there appear to be trends in these relationships in terms of the regions of the lumbar spine. Previous studies have demonstrated that during sagittal flexion, upper lumbar segments typically move first [24], and have the greatest ranges of motion [39]. In this study, L2-3 and L3-4 were strongly positively correlated, suggesting that they tend to work in tandem. However, they were also both negatively correlated with the IV-RoMmax of L4-5 and its initial attainment rate. This suggests a compensatory function between them, and if it is assumed that instability results from reduced restraint, then it may be suggested that reduced motion at these upper levels could feasibly be a factor in promoting L4-5 instability as a consequence of motion stress transfer.

Relationships with IV-RoMmax were different for the lower lumbar segments. While the ranges of L2-3 and L4-5 were inversely correlated, lordosis was positively associated with L2-3 range and negatively with L4-L5 range, while initial attainment rate at L4-5 was positively correlated with L4-L5 range, but negatively with the range of L2-3. This supports the view that a degree of lordosis may allow a more even sharing of motion throughout the lumbar spine, offering a degree of protection to the L4-5 segment during bending [26], and that lordosis itself has an important role in spinal biomechanical behaviour [40]. Together, these may have implications for prognosis in patients with L4-5 pain generation, a segment commonly involved in lumbar degeneration [22], especially if there is both hypo-lordosis and motion restriction in the upper lumbar spine.

Several relationships approached significance and may therefore also be important. L5-S1 and L4-5 IV-RoMmax and their initial attainment rates were positively correlated. However, this was not true for the upper segments. Increased range and initial attainment rate at L4-5 were both correlated to stiffness at L2-3 and added to by a reduction in lumbar lordosis, which has implications for proposed surgical stabilisation of upper levels. Conversely, while attainment rate and IV-RoMmax at L4-5 were positively correlated, L4-5 attainment rate was negatively correlated with the IV-RoMmax at L2-3 and with L3-4 above. As both attainment rate and IV-RoMmax are expressions of intervertebral restraint, these relationships may be regarded as compensatory, contributing to the attenuation of stress throughout the lumbar spine linkages. Thus there are indications of interactions and effects between kinematic and morphological variables at different levels.

Finally, there is an increasing awareness of the importance of sagittal parameters when planning surgical strategy [41, 42], correcting sagittal balance, or when considering more conservative treatment options. The ability to accurately assess and measure sagittal kinematic and morphological values may be important, as we attempt to understand their potential clinical utility [43]. The existence of intrinsic links between morphological variables such as lordosis have been described before [44], however we are the first to use continuous in vivo inter-vertebral motion to investigate its links with IV-RoMmax and initial attainment rate. These results provide clues as to what may happen when kinematic or morphological changes are imposed through conservative treatment or surgery, both as local and regional effects. The apparent inter-dependency may assist in building rationales for treatments, and highlights the need to account for factors such as lordosis when conducting kinematic studies. If the results are re-affirmed by multivariate investigations in larger samples, future longitudinal studies are recommended to investigate the effect of interventions in low back pain populations, that have been informed by the relationships described in this study.

### Limitations

The study’s results are only representative of one young, healthy, male population and replication with larger and more extensive populations would be required to explore the relationships in wider age groups and in females. In light of this, any discussions relating to the investigation and management of wider LBP populations warrant careful consideration. Furthermore, it was also not possible to address the impact of loading on spinal behaviour, although every effort was made to standardise the population sample and study protocol for body mass index. In this research all measurements were made during weight-bearing, and therefore the effect of muscle activity is also a consideration. A concurrent study conducted by our research group examines the relationships between lumbar paraspinal muscle activity and the kinematic and morphological variables described here [45]. Future studies may also wish to consider the use of dynamic stereo x-ray imaging [15], especially if investigation of rotation in the transverse or coronal planes is required, where associated out of plane movements are more prominent.

## Conclusions

IV-RoMmax and initial attainment rate measurements made using a QF weight-bearing sagittal plane protocol demonstrated acceptable reliability and agreement. Significant correlations were found between IV-RoMmax, IV-RoMmax at different inter-vertebral levels, lordosis and initial attainment rate. The study demonstrated weak to moderate effects of these variables on IV-RoMmax. The potential prognostic and treatment effects of these relationships merit exploration with multivariate studies in larger samples, potentially leading to longitudinal investigations in back pain populations.

## Additional file

Additional file 1: STROBE Statement—checklist of items that should be included in reports of observational studies (DOCX 40 kb)

## Abbreviations

AECC: Anglo-European College of Chiropractic
BMI: body mass index
CI: confidence interval
IBM SPSS: International Business Machines Corporation Statistical Package for the Social Sciences
ICC: intraclass correlation coefficient
IV-RoM: inter-vertebral range of motion
IV-RoMmax: maximum inter-vertebral range of motion
MRI: magnetic resonance imaging
mSv: millisievert
n: number
NRES: National Research Ethics Service
QF: quantitative fluoroscopy
SD: standard deviation
SEM: standard error of measurement
